# Natural history of fibrodysplasia ossificans progressiva: cross-sectional analysis of annotated baseline phenotypes

**DOI:** 10.1186/s13023-019-1068-7

**Published:** 2019-05-03

**Authors:** Robert J. Pignolo, Geneviève Baujat, Matthew A. Brown, Carmen De Cunto, Maja DiRocco, Edward C. Hsiao, Richard Keen, Mona Al Mukaddam, Kim-Hanh Le Quan Sang, Amy Wilson, Barbara White, Donna R. Grogan, Frederick S. Kaplan

**Affiliations:** 1 Department of Medicine, Mayo Clinic, Rochester, MN USA; 2 Departement de Genetique, Institut IMAGINE and Hôpital Necker-Enfants Malades, Paris, France; 3 Institute of Health and Biomedical Innovation (IHBI), Translational Research Institute, Princess Alexandra Hospital, Queensland University of Technology (QUT), Queensland, Australia; 4 Pediatric Rheumatology Section, Department of Pediatrics, Hospital Italiano de Buenos Aires, Buenos Aires, Argentina; 5 Unit of Rare Diseases, Department of Pediatrics, Giannina Gaslini Institute, Genoa, Italy; 6 Division of Endocrinology and Metabolism, the UCSF Metabolic Bone Clinic, the Institute of Human Genetics, and the UCSF Program in Craniofacial Biology, Department of Medicine, University of California-San Francisco, San Francisco, California, USA; 7 Centre for Metabolic Bone Disease, Royal National Orthopaedic Hospital, Stanmore, UK; 8 Departments of Medicine and Orthopaedic Surgery, The Center for Research in FOP and Related Disorders, Perelman School of Medicine, University of Pennsylvania, Philadelphia, PA USA; 9 Clementia Pharmaceuticals Inc, Newton, MA USA; 10 Departments of Orthopaedic Surgery & Medicine, The Center for Research in FOP and Related Disorders, Perelman School of Medicine, University of Pennsylvania, Philadelphia, PA USA

**Keywords:** Fibrodysplasia Ossificans Progressiva, Heterotopic ossification, Disease progression, Natural history, Clinical trial endpoints, Cross-sectional analysis

## Abstract

**Background:**

Fibrodysplasia Ossificans Progressiva (FOP; OMIM#135100) is an ultra-rare, severely disabling genetic disease characterized by congenital malformation of the great toes and progressive heterotopic ossification (HO) in muscles, tendons, ligaments, fascia, and aponeuroses often preceded by painful, recurrent soft tissue swelling (flare-ups). The formation of HO leads to progressive disability, severe functional limitations in joint mobility, and to a shortened life-span. In this prospective natural history study, we describe the baseline, cross-sectional disease phenotype of 114 individuals with FOP.

**Methods:**

All subjects underwent protocol-specified baseline assessments to determine their disease status. Cross-sectional analyses were performed using linear regression in which functional evaluations (Cumulative Analogue Joint Involvement Scale [CAJIS] and the FOP-Physical Function Questionnaire [FOP-PFQ]) and the burden of HO as measured by low-dose whole body CT (volume of HO and number of body regions with HO) were assessed.

**Results:**

Findings from 114 subjects (age range 4 to 56 years) were evaluated. While subject age was significantly (*p* < 0.0001) correlated with increased CAJIS (*r* = 0.66) and FOP-PFQ scores (*r* = 0.41), the estimated mean increases per year (based on cross-sectional average changes over time) were small (0.47 units and 1.2%, respectively). There was also a significant (*p* < 0.0001) correlation between baseline age and HO volume (*r* = 0.56), with an estimated mean increase of 25,574 mm^3^/year. There were significant (*p* < 0.0001) correlations between the objective assessment of HO volume and clinical assessments of CAJIS (*r* = 0.57) and FOP-PFQ (*r* = 0.52).

**Conclusions:**

Based on the cross-sectional analysis of the baseline data, functional and physical disability as assessed by CAJIS and the FOP-PFQ increased over time. Although longitudinal data are not yet available, the cross-sectional analyses suggest that CAJIS and FOP-PFQ are not sensitive to detect substantial progression over a 1- to 2-year period. Future evaluation of longitudinal data will test this hypothesis. The statistically significant correlations between HO volume and the functional endpoints, and the estimated average annual increase in total HO volume, suggest that the formation of new HO will be measurable over the relative short-term course of a clinical trial, and represents an endpoint that is clinically meaningful to patients.

**Trial registration:**

This study (NCT02322255) was first posted on 23 December, 2014.

**Electronic supplementary material:**

The online version of this article (10.1186/s13023-019-1068-7) contains supplementary material, which is available to authorized users.

## Background

Fibrodysplasia Ossificans Progressiva (FOP) (OMIM #135100) is a rare, severely disabling disease characterized by malformed big toes and progressive heterotopic ossification (HO) in muscles, tendons, and ligaments, and is often associated with painful, recurrent episodes of soft tissue swelling (flare-ups). FOP is caused by a recurrent heterozygous activating mutation of activin receptor A type I (ACVR1), a bone morphogenetic protein (BMP) type I receptor [1, 2]. There are approximately 800 confirmed cases of FOP globally [3] with an estimated prevalence of 0.6–1.3 per million individuals [4–6]. No available therapies have been demonstrated to prevent the formation of HO. Palliative treatment to alleviate symptoms is the current standard of care [7].

A classic feature of FOP is the formation of HO, often in the context of patient-reported flare-up symptoms of unpredictable frequency, duration, and location. Disease progression is also reported in the absence of flare-ups [8]. It is well recognized that recurrent episodes of HO formation starting in childhood lead to cumulative disability and functional limitations over the disease course, as well as to a shortened life span [9]. These insights into the natural history of FOP are derived from published case series [9–12] and a comprehensive global survey of 500 patients with FOP [8]. Unlike in earlier studies, all participants enrolled in this natural history study (NHS) of FOP had prospective, protocol-specified assessments of their disease at pre-specified time points.

This report describes the key design features of the NHS and the analysis of baseline cross-sectional data that describe the disease phenotype and potential endpoints with which to evaluate therapeutic candidates.

## Methods

The NHS is an on-going prospective, longitudinal, global, non-interventional study of male and female subjects clinically diagnosed with FOP due to the ACVR1 R206H mutation. The study is being conducted at seven international clinical sites (Buenos Aires, Argentina; Woolloongabba, Australia; Paris, France; Genoa, Italy; Stanmore, United Kingdom; Philadelphia, Pennsylvania, United States; and San Francisco, California, United States). Subjects are informed about the study through international and local patient organizations, direct physician outreach, and through postings on Clinicaltrials.gov and other similar websites.

The enrollment period began in December 2014 and ended in December 2016. The end of the planned 36-month follow-up for all subjects is expected in December 2019. All study sites obtained approval from their local institutional review boards and complied with all applicable national, local, ethical, and regulatory guidelines. All subjects, or minor subjects’ parents/legal guardians, were required to provide written informed consent. Age-appropriate assent was also obtained per local regulations. The study is registered on Clinicaltrials.gov (NCT02322255) and is sponsored by Clementia Pharmaceuticals Inc.

### Objectives

There are three overall objectives in the NHS: (1) describe the baseline FOP disease characteristics in order to identify clinically meaningful variables of disease progression that may serve as appropriate endpoints with which to assess the effectiveness of potential disease-modifying therapeutics; (2) measure FOP disease progression over 36 months of observation; and (3) systematically evaluate flare-up outcomes. This report describes the baseline data that support the first objective. Subsequent reports will present the results that support the latter objectives, when those data become available.

### Subject population and eligibility

Males and females from birth through 65 years of age, clinically diagnosed with FOP, and with verified ACVR1 R206H mutation (via centralized laboratory) were eligible for inclusion.

### Study design and timing of assessments

After screening and determination of eligibility, all subjects underwent a thorough baseline examination, including whole body computed tomography (WBCT) imaging, to determine their current disease status. The planned routine assessments over the 36-month observation period are shown in Table 1.

**Table 1:** Timing of standard assessments over the 3-year study

Assessment/Procedure	Screening/Baseline	First 3 weeks	Every 3 months	Every 6 months	Every 12 months
Clinic visit	X				X
Telephone contact		X	X	X	
Informed consent/assent	X				
Assess for eligibility	X				
Medical and FOP history	X				
Prior/concomitant medications	X	X	X	X	X
Post-baseline medical events				X	X
Physical examination	X				X
Linear height (≥ 18 years old)	X				X
Knee and sitting height (< 18 years old)	X				X
Body weight	X				X
Vital signs	X				X
Electrocardiogram	X				X
Pulmonary function tests	X				X
Pulse oximetry	X				X
Clinical laboratory assessments^a^	X				X
Blood and urine for biomarkers samples^b^	X				X
CAJIS	X				X
Knee and hand/wrist x-rays (< 18 years old)					X
WBCT (excluding head)	X				X
FOP assistive devices assessment	X			X	X
FOP-PFQ assessment	X			X	X
PROMIS Global Scales	X			X	X
Columbia-Suicide Severity Rating Scale	X				X
Genotyping	X				
Adverse events		X	X	X	X

^a^Includes triglycerides, alanine aminotransferase, aspartate aminotransferase, amylase, total cholesterol, lipase, total bilirubin, alkaline phosphatase, hemoglobin, hematocrit, platelets, and white blood cell count

^b^Bone and cartilage biomarkers include osteocalcin, bone specific alkaline phosphatase, P1CP-C-terminal propeptide of type 1 procollagen, P1NP N-terminal propeptide of type 1 procollagen, cartilage-derived retinoic acid-sensitive protein, and c-terminal telopeptide. The angiogenesis biomarker was urinary basic fibroblast growth factor. The inflammation biomarkers included erythrocyte sedimentation rate, c-reactive protein, interleukin-6, interleukin-1 beta, tumor necrosis factor-alpha, creatinine phosphokinase, and lactate dehydrogenase

Abbreviations: *CAJIS* = Cumulative Analogue Joint Involvement Scale, *FOP* = Fibrodysplasia Ossificans Progressiva, *FOP-PFQ* = Fibrodysplasia Ossificans Progressiva-Patient Function Questionnaire, *PROMIS* = Patient-Reported Outcome Measure Information System, *WBCT* = whole body computed tomography

### Endpoints

After screening and determination of eligibility, key endpoints were evaluated in all subjects to determine their baseline disease status.

Demographics (age, sex) and baseline flare-up characteristics (age at first flare-up, time since last flare-up, and number of flare-ups in the past 12 months) were reported. Blood and urine were also obtained for analysis of clinical laboratory parameters and potential biomarker activity (specific analytes are listed in Table 1).

The total body burden of HO was assessed by low dose WBCT, excluding the head. WBCT scout views were acquired in coronal and sagittal planes. WBCT scans were acquired in the cranio-caudal direction from the base of the skull through the feet using 3-mm axial slices with 512 × 512 matrix and pitch of one. Bone and soft-tissue kernels were utilized and coronal and sagittal reconstructions were generated. A single independent musculoskeletal radiologist at a central imaging laboratory used standardized procedures to review all baseline WBCT images to determine the presence/absence of HO across 15 body regions (neck, lower spine/abdomen, upper spine/chest; and left and right shoulders, elbows, wrists, hips, knees, and ankles). To determine total HO volume, HO was segmented on each axial slice using semi-automated seed growing and shrink wrap segmentation algorithms whenever possible. When not possible, manual contouring and nudging steps (Alice v9.0, PAREXEL Informatics, Waltham, MA) were used to optimize the HO segmentations as needed by the radiologist. The HO volumes were calculated separately for each of the 15 body regions and summed for the whole body burden of HO volume.

Range of motion across 12 joints (left and right shoulders, elbows, wrists, hips, knees, and ankles) and three body regions (jaw, cervical spine [neck], and thoraco-lumbar spine) was assessed using the Cumulative Analogue Joint Involvement Scale (CAJIS) for FOP [13]. Each joint/region was noted as: 0 = uninvolved; 1 = partially involved; 2 = completely ankylosed. Total scores ranged from 0 to 30, with higher scores indicating more severe limitations in mobility and function.

Physical function by subject report was assessed using the FOP-Physical Function Questionnaire (PFQ), a disease-specific instrument developed on the principles outlined in the FDA Guidance for Industry, “Patient-Reported Outcome Measures: Use in Medical Product Development to Support Labeling Claims” [14]. The instrument includes questions related to activities of daily living and physical functioning. Age-appropriate forms of the FOP-PFQ were completed by adults (subjects 15 years of age and older) and children/proxies (subjects 14 years of age and younger). Because the total scores vary by age across the FOP-PFQ instruments, the analysis was performed on transformed scores expressed as a percentage of the worst possible score with lower percentages indication worse functioning.

Physical and mental health were assessed using the Patient Reported Outcome Measure Information System (PROMIS) Global Physical and Global Mental Health Scales for subjects 15 years and older [15], and the PROMIS Pediatric Global Health Scale (proxy- and/or self-completed forms) for subjects 14 years of age and younger [16]. Scores were converted to T-scores such that a value of 50 (with a standard deviation of 10) represents the average for the general population in the United States. Higher T-scores indicate better physical/mental health.

## Statistical analysis

### Sample size

The sample size, which was based on enrollment projections throughout the global FOP community and not on statistical justifications, was set at up to 100 subjects (noting that subjects could be replaced at the discretion of the sponsor should they withdraw for any reason), with at least 10 subjects in each of the following categories: < 8 years old, 8 to < 15 years old, 15 to < 25 years old, and 25 to ≤65 years old. These age categories were chosen to obtain a representative cross-section of disease severity/progression.

### Planned analyses

The cross-sectional analysis of baseline data from all enrolled subjects was performed after the last subject was enrolled into the study. Data were tabulated descriptively (number and percentage of subjects for categorical parameters, and the number, mean, standard deviation, and range for continuous parameters) overall and by age category. The estimated cross-sectional difference over age in functional outcomes (FOP-PFQ and CAJIS) and the total body burden of HO as assessed by WBCT (volume of HO and number of body regions with HO) was assessed using linear regression with baseline age as the only covariate. Similarly, correlations between functional outcomes and the total body burden of HO were estimated with linear regression with measures of total body burden of HO as the only covariate. In focusing on one time point for each subject, these cross-sectional analyses do not take into account the episodic and variable nature of FOP progression. Correction for multiple testing was not performed.

## Results

### Demographics and baseline disease

The two sites in the United States enrolled the most subjects (22 [19%] in Philadelphia, PA; and 20 [18%] in San Francisco, CA), followed by Argentina (20 [18%] subjects), United Kingdom (17 [15%] subjects), France (15 [13%] subjects), Italy (14 [12%] subjects), and Australia (6 [5%] subjects).

Of the 117 subjects screened, 114 (97%) were documented to have the ACVR1 R206H mutation (two had variants and one did not have FOP; these subjects were not eligible for enrollment). The demographics and baseline disease characteristics of these 114 subjects (from 24 countries) are shown in Table 2.

**Table 2:** Demographics and baseline disease by age category

	< 8 Yrs (*N* = 17)	8 to < 15 Yrs (*N* = 36)	15 to < 25 Yrs (*N* = 34)	≥ 25 to ≤ 65 Yrs (*N* = 27)	Total (*N* = 114)
Males, *n* (%)	9 (52.9)	24 (66.7)	16 (47.1)	13 (48.1)	62 (54.4)
Age (years)					
Mean ± SD	5.9 ± 1.1	11.4 ± 2.1	18.9 ± 3.1	31.7 ± 6.7	17.6 ± 9.7
Median (min, max)	6.0 (4, 7)	11.0 (8, 14)	18.5 (15, 24)	30.0 (25, 56)	15.0 (4, 56)
Age at 1st flare-up					
Mean ± SD	2.9 ± 2.1	4.4 ± 3.6	5.6 ± 4.8	7.1 ± 5.0	5.2 ± 4.4
Median (min, max)	2.0 (1, 6)	4.0 (0, 13)	3.5 (0, 17)	5.0 (0, 20)	4.0 (0, 20)
Years since last flare-up					
Mean ± SD	0.7 ± 0.9	1.5 ± 2.8	1.5 ± 1.9	2.3 ± 3.5	1.6 ± 2.6
Median (min, max)	0.3 (0, 3)	0.5 (0, 14)	0.7 (0, 7)	0.9 (0, 15)	0.5 (0, 15)
Number of flare-ups in the past 12 months					
Mean ± SD	2.9 ± 2.6	6.8 ± 11.2	2.2 ± 1.8	1.9 ± 1.6	3.8 ± 6.9
Median (min, max)	2.0 (1, 10)	2.0 (1, 40)	1.0 (1, 8)	1.0 (1, 7)	2.0 (1, 40)
CAJIS total score^a^					
Mean ± SD	5.6 ± 3.9	9.2 ± 4.6	13.9 ± 6.7	16.5 ± 7.3	11.8 ± 7.0
Median (min, max)	6.0 (1, 15)	8.5 (1, 20)	13.5 (1, 26)	18.0 (1, 30)	10.5 (1, 30)
FOP-PFQ % worst total score^b^					
Mean ± SD	34.8 ± 26.1	44.4 ± 20.2	43.1 ± 31.0	58.4 ± 35.8	46.3 ± 27.1
Median (min, max)	41.8 (1, 84.6)	45.2 (1.9, 82.7)	38.4 (0, 100)	54.5 (0, 100)	45.5 (0, 100)
PROMIS Global Physical Health (adult) or PROMIS Global Health (pediatric, parent proxy) (T-score)^c^					
Mean ± SD	47.6 ± 9.4	41.8 ± 8.7	44.1 ± 9.0	42.5 ± 7.9	NA
Median (min, max)	48.3 (33, 24)	41.7 (24, 57)	44.9 (24, 68)	42.3 (27, 54)	
PROMIS Global Mental Health (adult) (T-score)^c^					
Mean ± SD	NA	NA	53.6 ± 9.9	51.4 ± 8.5	52.6 ± 9.3
Median (min, max)			53.3 (28, 68)	53.3 (39, 68)	53.3 (28, 68)
Total body HO volume, excluding head (mm^3^)					
Mean ± SD	61,951 ± 75,221	159,303 ± 161,779	380,751 ± 363,142	651,913 ± 674,454	324,631 ± 440,977
Median (min, max)	21,692 (0, 224,019)	130,509 (0, 828,262)	258,543 (0, 1,504,849)	481,524 (48,844, 2,833,946)	173,536 (0, 2,833,946)
Number of regions with HO^d^					
Mean ± SD	3.1 ± 2.6	5.1 ± 2.7	7.4 ± 3.4	8.8 ± 3.7	6.4 ± 3.7
Median (min, max)	4.0 (0, 7)	5.0 (0, 14)	8.0 (0, 13)	8.5 (3, 14)	6.0 (0, 14)

^a^CAJIS assessed range of motion across 12 joints (left and right shoulders, elbows, wrists, hips, knees, and ankles) and three body regions (jaw, cervical spine [neck], and thoraco-lumbar spine). Each noted as: 0 = uninvolved; 1 = partially involved; 2 = completely ankylosed. Total scores range from 0 to 30 with higher scores indicating more severe limitations in mobility

^b^FOP-PFQ used transformed scores expressed as a percentage of the worst possible score. Lower percentages indicate better functioning; higher percentages indicate worse functioning

^c^Distributions standardized such that a T-score of 50 (SD of 10) represents the average for the United States general population. Higher T-scores indicate better physical/mental health

^d^Fifteen possible regions (neck, lower spine/abdomen, upper spine/chest; and both shoulders, elbows, wrists, hips, knees, and ankles)

Abbreviations: *CAJIS* = Cumulative Analogue Joint Involvement Scale, *HO* = heterotopic ossification, *max* = maximum, *min* = minimum, *FOP-PFQ* = Fibrodysplasia Ossificans Progressiva-Patient Function Questionnaire, *NA* = not applicable, *PROMIS* = Patient-Reported Outcome Measure Information System, *SD* = standard deviation, *Yrs* = years

There was similar representation across the age categories (14.9% in the < 8 years group, 31.6% in the 8 to < 15 years group, 29.8% in the 15 to < 25 years group, 23.7% in the ≥25 to ≤65 years group). The mean age of subjects was 17.6 years (ranging from 4 to 56 years; median 15 years), with a slightly higher percentage of males (54.4%) than females (45.6%).

Retrospective report of flare-ups within the preceding 12 months tended to be greater in younger than in older subjects: the mean number of flare-ups reported in the previous 12 months was highest in the 8 to < 15 years group (6.8/year; median of 2.0/year) and lowest in the oldest group (1.9/year; median of 1.0/year); and the average time since a subject’s prior flare-up was 0.7 years (median of 0.3 years) in the youngest group compared with 2.3 years (median of 0.9 years) in the oldest group.

In general, mean baseline clinical laboratory and biomarker analytes were within the normal range for each age category.

Functional limitations, as assessed by the CAJIS and FOP-PFQ were worse in older than in younger subjects. Adult assessments of physical (PROMIS Global Physical Health) and mental (PROMIS Global Mental Health Scale) health were similar between the two older age categories. Children’s health as assessed by parent proxies on the PROMIS Global Health Scale was worse in older children than in younger children. The results are summarized in Table 2.

While the total body volume of HO was variable across the age groups (ranging from 0 to 2,833,946 mm^3^; Table 2), the median volume was lowest (21,692 mm^3^) in the youngest subjects and highest (481,524 mm^3^) in the oldest. The mean number of body regions with HO also increased with age, varying from 3.1 regions in the youngest age group to 8.8 regions in the oldest age group.

### Functional disability progresses as subjects age

**Fig. 1:**
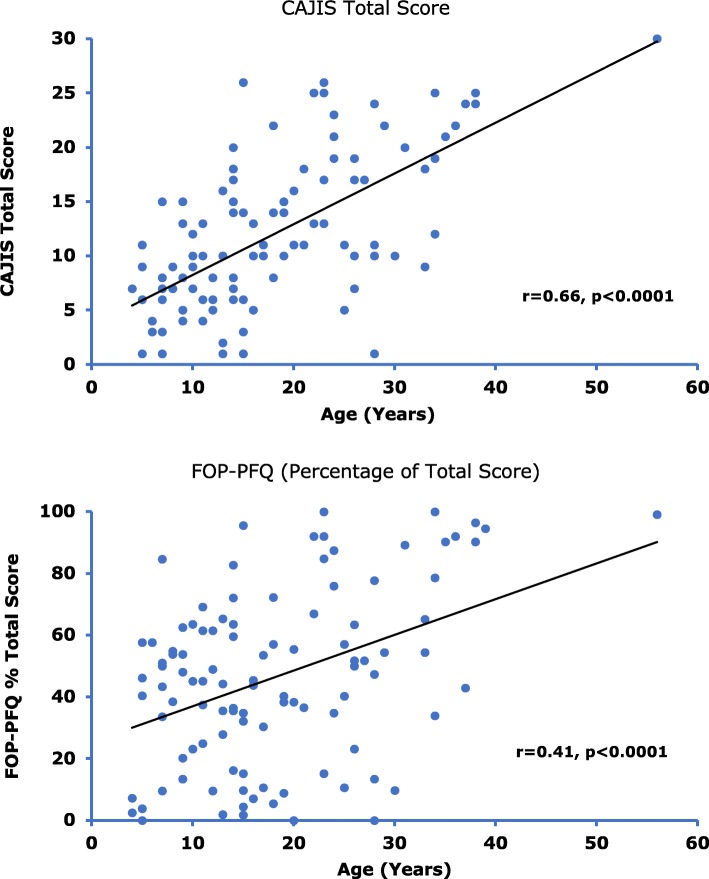
Correlation between Subject Age and Measures of Functional Disability. Correlation analysis of CAJIS Total Score and age (top) and FOP-PFQ Percent Total Score and age (bottom) in subjects with FOP. Correlation assessed using linear regression with baseline age as a covariate

Subject age was strongly correlated with CAJIS, and moderately correlated with the FOP-PFQ (Fig. 1), indicating that functional disability in FOP advances over time. The estimated average increases per year based on a linear regression model (0.47 units [95% CI: 0.37–0.57] for CAJIS and 1.2% [95% CI: 0.6–1.7%] for the FOP-PFQ) were relatively small for both assessments.

**Fig. 2:**
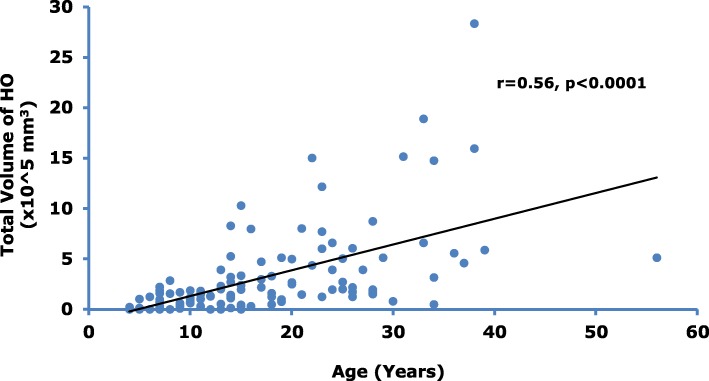
Correlation between Subject Age and HO Volume. Correlation analysis of total body volume of HO and age in subjects with FOP. Correlation assessed using linear regression with baseline age as a covariate

Subject age correlated with the total volume of HO (Fig. 2) and number of body regions with HO (Additional file 1: Figure S1), indicating that HO increases as subjects age. The average estimated increase in total HO volume was 25,574 (95% CI: 18445–32,704) mm^3^ per year of age; the average increase in the number of affected body regions was 0.22 (95% CI: 0.16–0.28) regions per year of age.

**Fig. 3:**
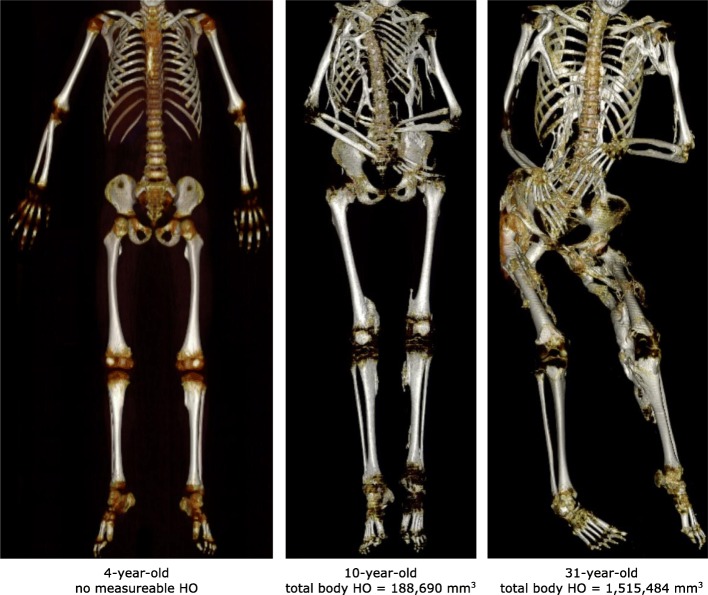
Whole Body Computed Tomography Images from Representative Subjects

A visual representation of quantity of HO is shown in the reconstructed WBCT scans from three representative NHS subjects of different ages (Fig. 3).

### HO burden correlates with clinical outcome measures

**Fig. 4:**
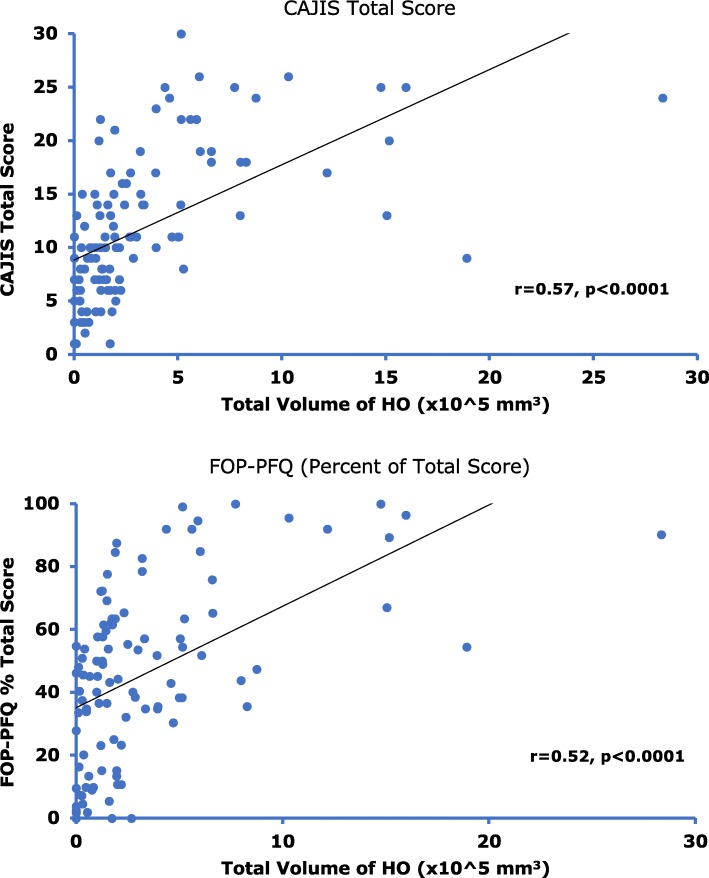
Correlation between Total Body Volume of HO and Measures of Functional Disability. Correlation analysis of CAJIS Total Score and volume of total body HO (top) and FOP-PFQ Percent Total Score and volume of total body HO (bottom) in subjects with FOP. Correlation assessed using linear regression with baseline age as a covariate

There was a moderate correlation between the volume of HO and CAJIS (*r* = 0.57, Fig. 4), and a strong correlation between the total number of body regions with HO and CAJIS (*r* = 0.72, Additional file 1: Figure S1). The correlations between the volume of HO and the FOP-PFQ (*r* = 0.52, Fig. 4), and between the total number of body regions with HO and the FOP-PFQ (*r* = 0.69, Additional file 2: Figure S2) were similar. These correlations indicate that HO substantially contributes to the functional limitations and disability that patients with FOP experience.

**Fig. 5:**
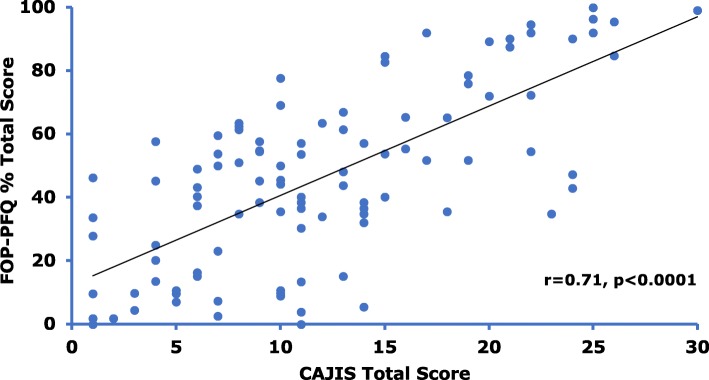
Correlation between CAJIS and FOP-PFQ. Correlation analysis of CAJIS Total Score and FOP-PFQ Percent Total Score in subjects with FOP. Correlation assessed using linear regression with baseline age as a covariate

The CAJIS and FOP-PFQ are also strongly correlated with each other (r = 0.71, *p* < 0.0001; Fig. 5) indicating that objective worsening in physical disability as assessed by the investigator closely align with subjective patient reports of functional impairment.

## Discussion

The challenges for the development of therapeutics to treat rare diseases include the limited number of patients available for study, the difficulty of demonstrating statistical significance with small samples, variability and uncertainty about disease progression and clinical outcomes, and the lack of established endpoints and validated instruments with which to measure progression [17]. Comprehensive natural history studies such as this one, when conducted in a prospective and systematic manner, can overcome many of these difficulties by identifying clinically meaningful and sensitive endpoints with which to assess potential disease-modifying agents.

In this international cohort of 114 subjects with FOP (representing approximately 13% of the known world-wide population of patients) [3], disease characteristics such as FOP onset and flare-up frequency, as well as the age and sex of the sample studied, were consistent with earlier retrospective studies that obtained patient information through survey and chart reviews [9, 18], mailed questionnaires [10], or anecdotal reports [12]. The flare-up findings were also similar to those reported in a 78-question flare-up survey of 500 FOP patients from 45 countries [8]. Given the size and composition of the NHS sample, and the consistent findings with earlier studies, the more detailed and prospective results from this NHS are likely to be representative of the FOP population worldwide.

Unlike earlier studies, a number of bone/cartilage, angiogenesis, and inflammation biomarkers were assessed at baseline. These will be followed long-term to determine whether any are predictive of FOP disease progression.

Subject age was significantly correlated with the CAJIS and the FOP-PFQ, suggesting that these instruments can measure the long-term progression of mobility and functional limitations as assessed by the physician and the patient. The small estimated average increases per year of 0.47 units for the CAJIS (similar to the estimated annual change of 0.5 units observed by Kaplan, et al) [13] and 1.2% for the FOP-PFQ indicate that a conclusive treatment benefit may not be demonstrable with either instrument over a 1- to 2-year timeframe of a typical clinical study. Evidence from the literature and clinician experience indicate that recurrent and cumulative episodes of HO formation, the pathognomonic feature of FOP, begin in childhood and lead to increasing disability and functional limitations over time. This is consistent with the significant correlations observed between the total body burden of HO (ie, volume of HO and the number of body regions with HO) and age. These results imply that potential treatments should target the pediatric population in order to prevent and/or minimize the irreversible disability that occurs as patients age.

The estimated average increase in total HO volume of 25,574 mm^3^/year suggests that new HO is an endpoint that will be measurable over the relative short-term course of a clinical study. The correlations between the total body burden of HO and the loss of movement (as assessed by range of motion across 15 different body regions in the CAJIS) and worsening functional impairment (as measured by the disease-specific FOP-PFQ) strongly suggest that HO is a clinically meaningful endpoint. As the number of regions with HO increases and/or the volume of HO increases, there is a commensurate decrease in mobility, including complete ankyloses of joints and increasing functional impairment over time. It should be noted that some subjects have a high degree of immobility (as assessed by CAJIS) or physical dysfunction (as assessed by the FOP-PFQ) but little measurable HO (Fig. 4). This may be due to small amounts of HO that are below the level of detection of the WBCT scans; the specific location of the HO that is impeding joint movement; or other factors such as congenital joint malformations or severe degenerative joint disease that may also contribute to loss of movement and function in a small percentage of subjects [19]. In addition, the variability of HO volume observed at baseline across the age groups will further challenge the investigation of potential therapeutics in FOP.

While the cross-sectional analyses of the baseline data from this NHS adds to the clinical perspective on FOP by quantitatively estimating the rate of progression of bone deposition and change in functional impairment over time, the results must be confirmed longitudinally. One of the objectives of the NHS is to obtain such long-term data on disease progression over 36 months. Thus, the assessments performed at baseline are being repeated annually and the reported estimated changes will be corroborated with the actual changes observed over time. Another limitation is that the NHS only enrolled patients with confirmed R206H mutation in the ACVR1 gene. However, this mutation is present in 97% of patients with FOP, and few patients have other FOP-causing mutations in this gene [20, 21].

## Conclusions

The baseline data obtained in this NHS are believed to be representative of the world-wide FOP population. These data contribute to our understanding of FOP by characterizing the cross-sectional changes in physical and functional impairment over the course of the disease and emphasizing the importance of HO as a substantial cause of morbidity. In addition, the results provide a rationale for the selection of endpoints that may be utilized in clinical studies of potential disease-modifying therapeutics in FOP. In particular, the total body burden of HO as assessed by WBCT as a clinically meaningful outcome measure is sufficiently sensitive to document estimated disease progression and treatment effects over 1–2 years. However, this needs to be verified in ongoing longitudinal studies in FOP patients. A therapeutic that reduces, relative to untreated subjects, the number of body regions with new HO, and/or decreases the formation of new HO volume, should change disease trajectory and prolong patients’ functional independence.

## Additional files

Additional file 1: **Figure S1.** Correlation between Measures of Functional Disability and Number of Regions with HO. Correlation analysis of CAJIS Total Score and age (top) and FOP-PFQ Percent Total Score and total number of body regions with HO (bottom) in subjects with FOP. Correlation assessed using linear regression with baseline age as a covariate. (PDF 48 kb)

Additional file 2: **Figure S2.** Correlation between Subject Age and Body Regions with HO. Correlation analysis of number of body regions with HO and age in subjects with FOP. Correlation assessed using linear regression with baseline age as a covariate. (PDF 46 kb)

## Abbreviations

ACVR1: Activin receptor type 1A
BMP: Bone morphogenetic protein
CAJIS: Cumulative Analog Joint Involvement Scale
CT: Computed tomography
FOP: Fibrodysplasia ossificans progressiva
FOP-PFQ: FOP-Physical Function Questionnaire
HO: Heterotopic ossification
NHS: Natural history study
OMIM: Online Mendelian Inheritance in Man
PROMIS: Patient Reported Outcome Measure Information System
WBCT: Whole body computed tomography
