# Correction to: Natural history of fibrodysplasia ossificans progressiva: cross-sectional analysis of annotated baseline phenotypes

**DOI:** 10.1186/s13023-019-1096-3

**Published:** 2019-05-23

**Authors:** Robert J. Pignolo, Geneviève Baujat, Matthew A. Brown, Carmen De Cunto, Maja Di Rocco, Edward C. Hsiao, Richard Keen, Mona Al Mukaddam, Kim-Hanh Le Quan Sang, Amy Wilson, Barbara White, Donna R. Grogan, Frederick S. Kaplan

**Affiliations:** 1 Department of Medicine, Mayo Clinic, Rochester, MN USA; 2 Departement de Genetique, Institut IMAGINE and Hôpital Necker-Enfants Malades, Paris, France; 3 Institute of Health and Biomedical Innovation (IHBI), Translational Research Institute, Princess Alexandra Hospital, Queensland University of Technology (QUT), Queensland, Australia; 4 Pediatric Rheumatology Section, Department of Pediatrics, Hospital Italiano de Buenos Aires, Buenos Aires, Argentina; 5 Unit of Rare Diseases, Department of Pediatrics, Giannina Gaslini Institute, Genoa, Italy; 6 Division of Endocrinology and Metabolism, the UCSF Metabolic Bone Clinic, The Institute of Human Genetics, and the UCSF Program in Craniofacial Biology, Department of Medicine, University of California-San Francisco, San Francisco, California USA; 7 Centre for Metabolic Bone Disease, Royal National Orthopaedic Hospital, Stanmore, UK; 8 Departments of Medicine and Orthopaedic Surgery, The Center for Research in FOP and Related Disorders, Perelman School of Medicine, University of Pennsylvania, Philadelphia, PA USA; 9 Clementia Pharmaceuticals Inc, Newton, MA USA; 10 Departments of Orthopaedic Surgery & Medicine, The Center for Research in FOP and Related Disorders, Perelman School of Medicine, University of Pennsylvania, Philadelphia, PA USA

**Corretion to: Orphanet J Rare Dis (2019) 14:98**

**https://doi.org/10.1186/s13023-019-1068-7**

The original version of this article [1] unfortunately included an error to an author’s name. Author Maja Di Rocco was erroneously presented as Maja DiRocco.

The correct author name has been included in the author list of this Correction article and is already updated in the original article.
