# Supplementary figures and images for: Natural history of fibrodysplasia ossificans progressiva: cross-sectional analysis of annotated baseline phenotypes
Source: Orphanet J Rare Dis. 2019 May 3;14:98. doi: 10.1186/s13023-019-1068-7 (PMC6499994; doi:10.1186/s13023-019-1068-7)

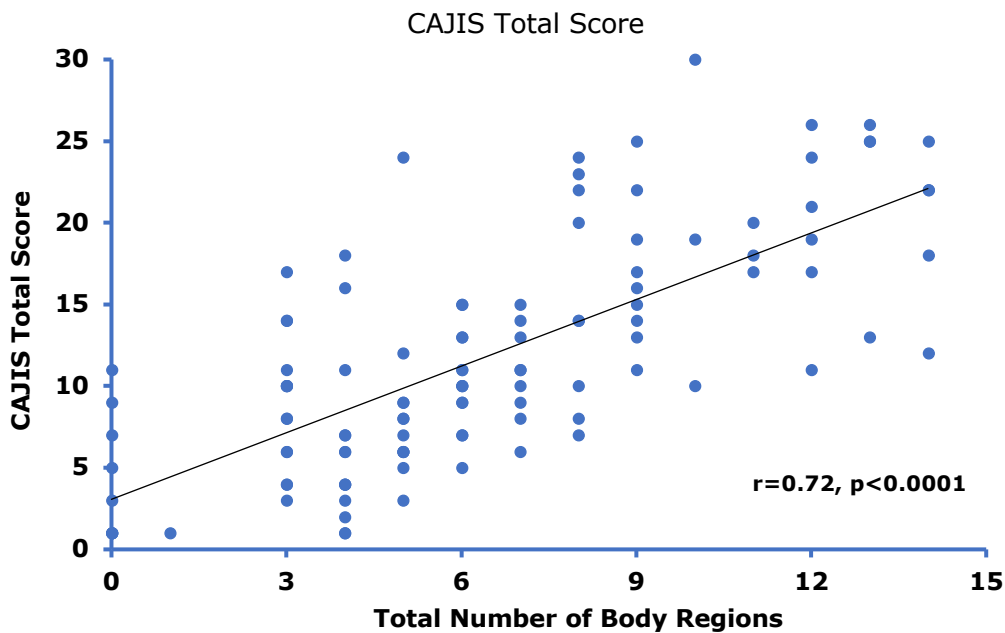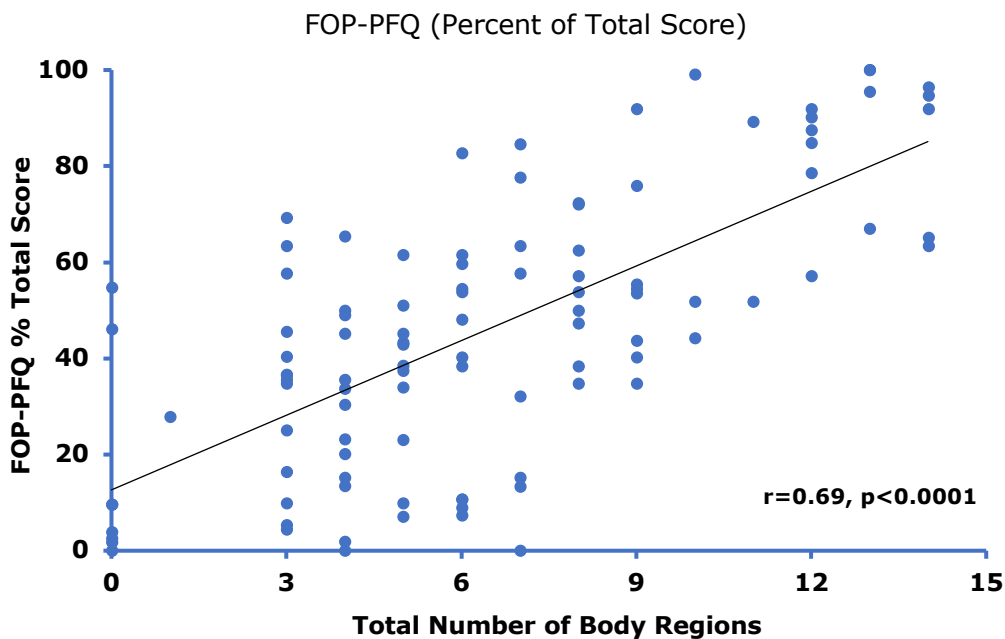

Supplement: Supplementary file 1 — Figure S1. Correlation between Measures of Functional Disability and Number of Regions with HO. Correlation analysis of CAJIS Total Score and age (top) and FOP-PFQ Percent Total Score and total number of body regions with HO (bottom) in subjects with FOP. Correlation assessed using linear regression with baseline age as a covariate. (PDF 48 kb) [file 13023_2019_1068_MOESM1_ESM.pdf]

Total Number of Regions

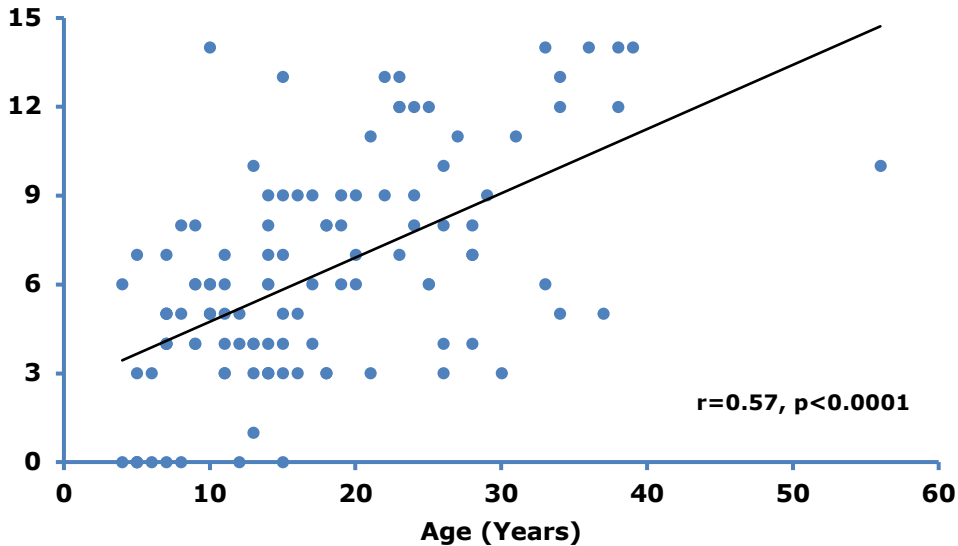

Supplement: Supplementary file 2 — Figure S2. Correlation between Subject Age and Body Regions with HO. Correlation analysis of number of body regions with HO and age in subjects with FOP. Correlation assessed using linear regression with baseline age as a covariate. (PDF 46 kb) [file 13023_2019_1068_MOESM2_ESM.pdf]
